# Novel Lines of Research on the Environmental and Human Health Impacts of Nut Consumption

**DOI:** 10.3390/nu15040955

**Published:** 2023-02-14

**Authors:** Linda Tapsell, Joan Sabaté, Raquel Martínez, Marc Llavanera, Elizabeth Neale, Albert Salas-Huetos

**Affiliations:** 1 School of Medical Indigenous and Health Sciences, University of Wollongong, Wollongong, NSW 2522, Australia; 2 Center for Nutrition, Lifestyle and Disease Prevention, School of Public Health, Loma Linda University, Loma Linda, CA 92350, USA; 3 Unit of Cell Biology, Department of Biology, Faculty of Sciences, University of Girona, 17003 Girona, Spain; 4 Biotechnology of Animal and Human Reproduction (TechnoSperm), Institute of Food and Agricultural Technology, University of Girona, 17003 Girona, Spain; 5 Unit of Preventive Medicine and Public Health, Faculty of Medicine and Health Sciences, Universitat Rovira i Virgili, 43201 Reus, Spain; 6 Department of Nutrition, Harvard T.H. Chan School of Public Health, Harvard University, Boston, MA 02115, USA; 7 Consorcio CIBER, M.P., Fisiopatología de la Obesidad y Nutrición (CIBERobn), Instituto de Salud Carlos III (ISCIII), 28029 Madrid, Spain

**Keywords:** nuts, environment, sustainability, reproduction, sexual function, diet, dietary patterns

## Abstract

Nuts have formed part of human diets throughout the ages. In recent decades, research has shown they are key foods in dietary patterns associated with lower chronic disease risk. The current state of climate change, however, has introduced an imperative to review the impact of dietary patterns on the environment with a shift to plant-based diets. Nuts emerge as a significant source of protein in plant-based diets and are a minimally processed and sustainable food. Research in this area is evolving to drive better production methods in varying climate conditions. Nevertheless, nut consumption remains an important contributor to human health. The mechanisms of action can be explained in terms of the nutrients they deliver. Studies of nut consumption have linked components such as monounsaturated fatty acids, plant omega-3 fatty acids, antioxidants, and plant sterols to improved lipoprotein profiles, lower blood pressure, and reduced cardiovascular disease risk. Preliminary research also indicates possible beneficial effects of nut consumption on reproductive health. In any case, the ultimate effects of foods on health are the results of multiple interactive factors, so where nuts fit within dietary patterns is a significant consideration for research translation. This has implications for research methodologies, including categorization within food groups and inclusion in Healthy Dietary Indices. The aim of this narrative review is to outline new focal points for investigation that examine the environmental and some novel human health impacts of nut consumption and discuss future directions for research.

## 1. Introduction

As naturally occurring edible and nutritious foods, nuts have been part of the human diet throughout the ages [1]. Modern nutrition science provides evidence of their health benefits, but foods have not always been the object of nutrition research, with a focus in past decades on nutrients contained in foods. At the same time, the industrialization of the food supply saw the emergence of chronic lifestyle-related diseases, such as obesity, cardiovascular disease (CVD), and type 2 diabetes. Research identified so-called ‘negative’ components of the diet including dietary fat and excess energy consumption [2], which translated to dietary advice and created an ambiguous position for nuts as a high-energy, high-fat food.

Population-based dietary guidelines appeared in the 1980s aimed at providing adequate nutrition and preventing chronic disease. Early guidelines referred to staple food groups, with advice to avoid foods high in fat, sugar, and salt [2]. The position of nuts in food groups was variable, but the value of naturally occurring foods, captured in the concept of food synergy [3], and an appreciation of the relationship between nutrients, foods, and dietary patterns [4] led to today’s guidelines having a greater focus on dietary patterns. Research on nuts followed this direction, expanding beyond their nutritional contributions to nuts as a significant food in healthy dietary patterns. Direct clinical evidence of health effects came from trials involving at-risk populations. Basic science research provided insights into the molecular pathways underlying health effects, and epidemiological studies confirmed that associations between nut consumption and health outcomes occurred in the broader population. Each of these types of research were important in building the body of evidence, despite challenges in providing timely and consistent studies to support nutrition policy and practice [5,6,7]. At the same time, clinical evidence review methodology has developed further to consider the quality as well as quantity of research. This development recognized that research practices and study designs require sufficient scrutiny to assure confidence in results and valid translation to practice [8].

Today, evidence supporting nut consumption is extensive. It goes beyond chronic disease prevention to other forms of human health—including reproductive health—and then to the planet’s health. The environmental impact of healthy dietary patterns is part of the evidence analysis, as nuts are significant foods in plant-based diets. The global imperative to address climate change calls for additional research methodologies that address the environmental impact of foods [8]. Today, there are strong calls to combine imperatives for human health with that of the environment [9].

The aim of this narrative review is to outline new focal points for research that examine the environmental and novel human health impacts of the consumption of nuts and discuss future directions.

## 2. Nuts and Environmental Sustainability

Environmental sustainability is one of the most pressing new areas of research on food today. Food production and consumption face unprecedented scrutiny as their impact on the natural environment and human health becomes more evident. How foods are grown (including water use), processed, sold, prepared, cooked, consumed, and disposed of is crucial. In studying nuts and environmental sustainability, all these stages of production and consumption (the ‘food system’) must be considered.

The global food system produces enough calories for a growing world population. However, about half of the population is malnourished with almost 1 billion people not consuming enough food and experiencing hunger, and almost 2 billion overconsuming foods low in nutritional quality associated with micronutrient and phytonutrient deficiencies, obesity, and increased incidence of chronic disease. Globally, the non-optimal intake of foods in the diet is estimated to account for approximately 22% of all deaths among adults and 15% of disability-adjusted life-years [10]. At the same time, agriculture uses ~70% of the global freshwater [11]. The food supply chain is responsible for ~26% of global greenhouse gas emissions (GHGe), occupies ~43% of habitable land, causes ~78% of the ocean and freshwater eutrophication, and ~32% of terrestrial acidification worldwide [12].

Several strategies have been proposed to decrease the environmental pressures exerted by the food systems. These include (1) improving agricultural technologies to enhance productivity and reduce harmful emissions; (2) reducing food loss and waste to decrease food production requirements and waste emissions in landfills; and (3) shifting to the production and consumption of foods that support human and planetary health [13,14,15,16]. Thus, the type and amount of food produced and consumed are major determining factors in promoting human health within planetary boundaries [17,18]. Research can identify where nuts fit within these parameters. Addressing the environmental impacts of the lifecycle of nuts (LCA, Lifecycle Analysis), from production to consumption, is one way to approach this.

**Figure 1:**
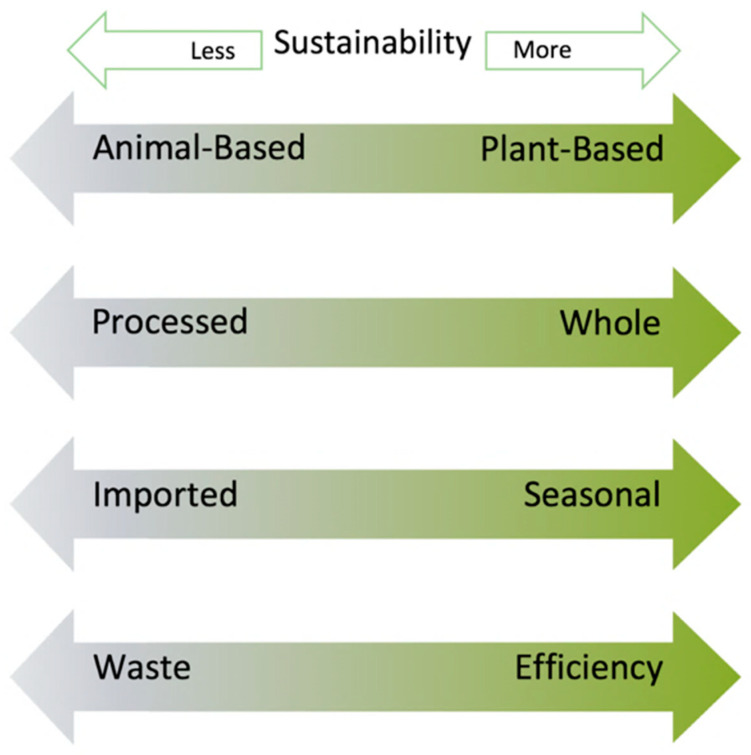
Characteristics of foods in a diet that determines its sustainability. The graphic illustrates that a sustainable diet has a higher proportion of foods that are plant-based, whole, in-season, and consumed with no or minimal waste [18].

According to the Food and Agriculture Organization (FAO), environmentally sustainable diets are “those diets with low environmental impacts which contribute to food and nutrition security and to healthy life for present and future generations (…) while optimizing natural and human resources [19].” We have previously identified four determinants for, or dimensions of, a sustainable diet from the consumer’s perspective. These dimensions are based on the ratios of dietary characteristics. They are (1) the proportion of foods in the diet of animal versus plant origin, (2) the proportion of processed versus whole foods, (3) the proportion of seasonal/locally sourced foods versus out-of-season/context, and (4) the proportion of foods consumed versus wasted [18] (Figure 1).

Most diets (and most meals) have a mix of foods, each having different characteristics. The inclusion of nuts in the diet is variable. The larger the amount and proportion of foods in a meal or diet whose constituent foods are animal-sourced, processed, out of season or context (requiring transportation or refrigeration for storage), and wasted, the less sustainable the diet is. Reciprocally, the higher the proportion of foods of plant origin consumed, minimally processed, in season, and locally sourced, the more sustainable the diet is. Thus, nuts would appear to have a place in sustainable diets.

### 2.1. Nuts as Sustainable Foods

At the consumption level, nuts are plant foods consumed whole or very minimally processed; they have a “long season” (since they do not require refrigeration for storage, can be transported and stored with minimal energy use), and have little waste. Thus, nuts appear to be sustainable foods.

At the production level, however, concerns have been raised regarding the high water usage and chemical inputs in the production of nuts under intensive agricultural practices. Importantly, this is not the case when nuts are grown in extensive or traditional agricultural practices.

#### 2.1.1. Life Cycle Analysis

There is a relative paucity of published data from Lifecycle Analysis (LCA) on nuts. Environmental impacts of foods can be measured using various units, including, per weight (of edible amount), per serving, or, depending on the nutritional contribution of each food, per grams of protein or energy (kcal) [20,21,22]. This can be problematic for some foods such as nuts. For example, many food comparisons regarding environmental impacts have been based on edible amounts (by weight), giving nuts mixed results. A commonly recommended nut serving size is small (approximately 30 g) compared to other foods (often 50–120 g or even 240 g for most beverages). However, nuts are also energy-dense foods (less than 5% water) whose main nutrients are fat and protein. As a result, the environmental impacts of certain nuts measured per grams of protein are very low compared to other animal-sourced protein-rich foods.

**Figure 2:**
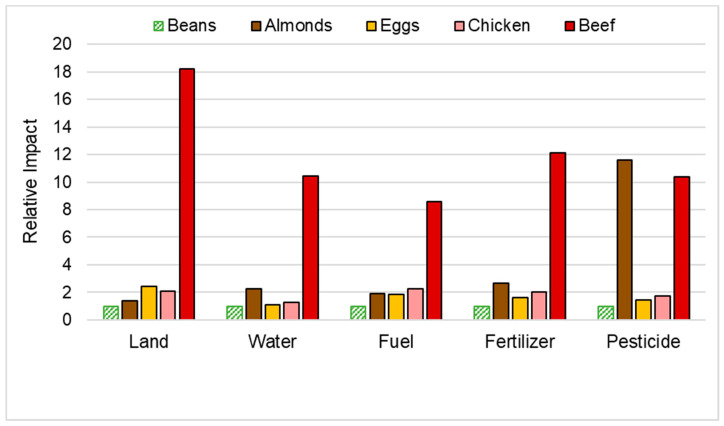
Relative Environmental Impacts of Protein Food Sources in relation to protein from beans [23].

We conducted a LCA of five common food sources of protein: legumes, nuts, eggs, poultry, and red meat—specifically, kidney beans (*Phaseolus* sp.), almonds (*Prunus dulcis*), eggs, chicken, and beef as produced in Californian agricultural practices [23]. When using beans as the reference (legumes are nature’s most efficient production of protein), almond protein is the second best ranked after beans for most environmental parameters except for pesticides (Figure 2).

Clark and colleagues [24] analyzed LCA data from meta-analyses to determine the impact of fifteen foods on environmental depreciation, encompassing five components: plausible acidification, eutrophication, GHGe, land use, and scarcity-weighted water use. Of the fifteen analyzed foods, red meat (100 g), chicken (100 g), eggs (50 g), legumes (50 g dried weight; DW), and nuts (28 g) represent protein sources [25]. Each food was depicted in a radar plot, illustrating the rank-ordered impingement on designated environmental parameters per daily food serving. When comparing nuts, eggs, and red meat, nuts performed relatively well on all environmental parameters except water use. The environmental impact per serving of eggs per day serves as an intermediate. Red meat received the highest or most detrimental rank in all five environmental parameters, thus corresponding with previous research [23].

The foods were rank-ordered from least to most environmentally impactful per serving produced [24]. Nuts ranked lowest (least harmful) for GHGe among all fifteen foods. Among the five protein food sources, nuts ranked lowest in eutrophication potential and second lowest in acidification and land use. However, in conjunction with previous scholarly [26] and media [27,28] critiques on nut production and water use, nuts ranked second highest in scarcity-weighted water use, only exceeded by red meat. These findings imply that the environmental impact of a 28 g serving of nuts is less aggravating than that of a 100 g serving of red meat, although water use is of concern.

#### 2.1.2. Water Footprint

The situation for nuts requires further examination of the term ‘water use’. This complex issue may be better addressed by considering distinct ‘water footprints’ referring to ‘blue water’ (surface plus groundwater, often used in irrigation [29]) and ‘green water’ (rainwater consumed with agricultural production [26]). Mekonnen and Hoekstra calculated the water footprints of nuts [26] and farm animals [30] from 1996 to 2005. Vanham and colleagues [31] applied the water footprint computations to demonstrate the blue and green water footprint of nuts and animal proteins (beef, eggs, chicken, pig meat, and sheep meat) in liters per kilogram and liters per gram of protein. In the context of liters per kilogram, shelled cashew (*Anacardium occidentale*) nuts have the most prominent combined blue and green water footprint among all nuts and triple that of beef. The water footprints of almonds and pistachios are also considerable, exceeding 10,000 L per kilogram. Among the selected animal proteins, eggs have the lowest water footprint. When comparing the water footprint by food weight, the sustainability of nuts appears unfavorable; however, as previously mentioned, protein is a sizable component of nuts’ nutrients, suggesting that water footprint expressed per gram of protein may be a more accurate representation for this environmental impact.

Further clarification on the position of different types of nuts is seen through the water footprint articulated in liters per gram of protein. Accordingly, the water footprint of cashews remains higher than that of beef, whereas that of peanuts is lower than all five animal proteins [31]. Moreover, the average green and blue water footprint of almonds, hazelnuts (*Corylus* sp.), pistachios (*Pistacia vera*), and walnuts (*Juglans* sp.), combined, remain lower than beef’s water footprint when based on liters per gram of protein. Comparing the environmental impact of foods based on protein content rather than weight may be valuable from a nutritional perspective, as eating patterns focus on nutrient levels rather than weight.

Researchers must also address the location of nut production, as agricultural production in water-scarce areas may have a greater impact on water footprint than locations with greater water availability. Indeed, water stress, defined as the ratio of water used to available water [32], varies according to region. For example, in California, the powerhouse of almond production [33], high water stress [31] and water demand [34] are evident even though the carbon footprint is relatively low [35]. Agricultural practices, including intensive versus extensive agriculture [36], require further consideration because these determine the magnitude of resource utilization.

### 2.2. Future Research on Nuts and Sustainability

First and foremost, future research should prioritize collecting data on nut production and sustainability using environmental parameters beyond water and GHGe. Although data regarding acidification and eutrophication potency are available [24], they are limited, and this confines the current comprehension of the environmental impact of producing nuts. Furthermore, collecting and comparing data on the environmental impact of nut production according to agricultural methods (intensive versus extensive), climate setting, and location may clarify whether previous critiques of nut production and sustainability are consistently reasonable across varied agricultural conditions.

Certainly, an increase in the production and consumption of nuts, as recommended by the EAT–Lancet Commission [9], a global initiative on food and planetary health, beckons the question of whether eating more nuts is more sustainable than “healthier” diets. If “healthier” diets were to incorporate conventional meat analogs, it would be necessary to compare their sustainability with that of nuts. This would require quantifying the LCA of nuts versus conventional meat analogs in isocaloric and isoprotein conditions. Additional modeling or use of diet records can be useful in describing the environmental impact of nuts in previously defined dietary patterns.

The future of nuts in the diet may even involve replacing well-known meat analogs, including texturized vegetable protein (TVP) [37], with nut-based meat analogs. Notably, this implies assessing and comparing the sustainability of nut-based meat analogs with current TVPs using the parameters discussed above.

In addition, nuts are an excellent source of fat, almost entirely unsaturated. They contain mainly monounsaturated fatty acids (MUFAs) and some polyunsaturated fatty acids (PUFAs), mostly *n*-6 PUFAs, while walnuts are a good source of vegetable *n*-3 PUFAs [38]. Given the importance of these essential and healthy fats, relevant research could include the computation of LCA environmental inputs relative to the different food sources of unsaturated fats, including *n*-3 PUFAs.

Unsustainable and unhealthy foods harm both planetary health and human well-being. Further research is required demonstrating and quantifying the environmental sustainability of nuts. Specifically, more needs to be known about the efficient use of natural resources and environmental protection in the production, preparation, and disposal of nuts. Consumption needs to be addressed in terms of nuts as a single food, as an alternative to other foods, and in the context of healthy dietary patterns.

## 3. Nuts and Male Reproductive Health

From environmental concerns to effects on human biology, research on the health effects of nuts has extended beyond the scope of chronic non-communicable diseases to the support of human growth and development. One particularly novel area is that of infertility, defined as the incapacity to conceive after one year or longer of unprotected intercourse. Infertility affects approximately 15% of the world’s population [39]. Male factors are responsible for 40–50% of infertility cases, and an evident decline in semen quality parameters has been reported over the last fifty years [40,41,42,43]. A recent meta-regression analysis, including 185 studies and more than 42,000 men without known fertility problems, estimated a 50–60% decrease in sperm counts between 1973 and 2011 [44]. Accordingly, despite the multicausal nature of male infertility, the examination of the factors negatively affecting semen quality is warranted to develop novel strategies to prevent, diagnose, and treat male infertility disorders [45]. Several etiologies of male infertility have been uncovered over the years, such as specific lifestyle factors, congenital/genetic disorders, hormonal imbalances, or sexually transmitted diseases [45,46]. Among all male (in)fertility-associated factors, lifestyle habits are modifiable factors highly associated with sperm quality, which plays a key role in reproductive health [47].

**Figure 3:**
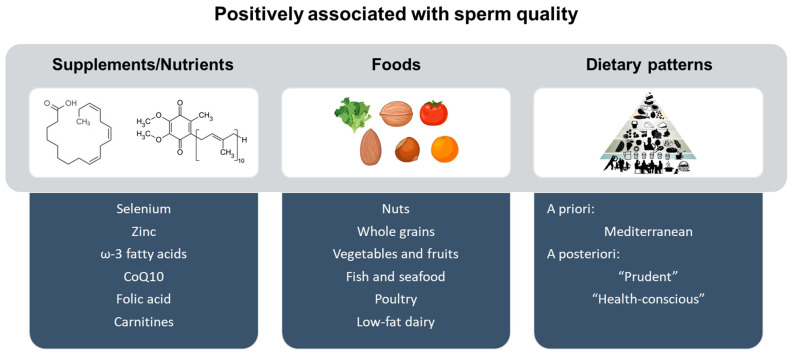
Supplement/nutrient intake, food consumption, and dietary pattern adherence positively associated with sperm quality parameters [61].

Dietary patterns, specific foods, and their nutrient components have been reported as essential factors for proper sperm function and male fertility [48]. The adherence to a healthy diet [49,50,51,52,53,54,55,56,57], including the consumption of fruits and vegetables, nuts, fish, seafood, and whole-grain cereals, while avoiding excessive intake of processed high-fat products, alcoholic beverages, caffeine, and sugary drinks [49,58,59,60,61] have been positively associated with sperm quality (Figure 3).

In this regard, adhering to unhealthy dietary habits could exert a negative impact on semen quality, thus impairing the function of male gametes and reducing or preventing fecundability [45]. This warrants the study of novel dietary habits contributing to improving sperm quality and thereby reducing male-factor infertility.

Recognized as a component of healthy dietary patterns, nuts are nutrient-dense foods rich in unsaturated fatty acids, fiber, minerals, vitamins (among them, tocopherols), phytosterols, polyphenols, and other antioxidants. Nuts deserve special attention for their potential role in male reproductive health, given the general beneficial impact on several health outcomes [38,62,63,64]. The next section of the review examines the potential impact of nut consumption on sperm quality and functionality, as well as on sexual function.

### 3.1. Nuts and Sperm Quality and Functionality

Animal models have demonstrated possible benefits of nut consumption for sperm quality and functionality. Two recent articles focused on the effects of hazelnut [65] and cashew [66] supplementation. In one study, Kara et al. randomized a group of rats into a control (ad libitum laboratory standard diet) and hazelnut-supplemented feed group, with a daily dose of 3 g hazelnut kg^−1^ bodyweight. The authors reported that the supplementation corresponded to 5.5% of hazelnut in the diet and equated to 30 g day^−1^ in humans. Following a thirty-day intervention, the inclusion of hazelnuts significantly improved sperm vitality. Moreover, the hazelnut intervention also significantly improved the Johnsen’s testicular histologic score, a system conventionally used to evaluate the completeness of testicular spermatogenesis, and reduced apoptotic indices [65]. In another study, Akomolafe et al. investigated the effect of cashew nut supplementation on fertility in rats. The rats were randomly divided into six diet groups with varying cashew nut (10 to 20%) and clomiphene citrate composition in the diets. The combination of cashew nut supplementation and clomiphene citrate significantly increased epididymal sperm count, vitality, and motility, and decreased total sperm abnormalities in comparison to the control [66]. These findings suggest that the inclusion of nuts (e.g., hazelnuts or cashews) may be useful to treat male partners suffering from sperm quality problems and infertility issues.

The effects of nut consumption on sperm quality have been tested in humans in two randomized clinical trials (RCT) [67,68]. Robbins and collaborators were the first to report substantial improvements in sperm vitality, motility, and morphology after the inclusion of 75 g day^−1^ of walnuts for 12 weeks by 117 healthy men following Western-style diets [67]. The authors attributed these improvements to the increase in blood *n*-6 PUFAs and alpha-linolenic acid (ALA), the vegetable *n*-3 PUFA, hypothesizing on potential mechanisms of action (ALA is a well-established biomarker of walnut consumption [69,70]). In a second study, the FERTINUTS trial, Salas-Huetos et al. evaluated the effect of chronic consumption of mixed nuts (almonds, hazelnuts, and walnuts) on changes in conventional semen parameters, implicating several potential mechanisms. A total of 119 healthy reproductive-age men consuming a Western-style diet were randomized and allocated to two diet groups, one enriched with 60 g mixed nuts day^−1^ and the other devoid of nuts. The inclusion of nuts significantly improved total sperm count and sperm cell vitality, motility, and morphology, and these findings were explained in terms of a reduction in sperm DNA fragmentation [68]. Nut consumption was also associated with a reduction in the micro-RNA hsa-miR-34b-3p expression level [68] and with differential methylation in 36 genomic regions between the baseline and the end of the trial [71]. These studies suggest that sperm epigenome mechanisms can respond to diet.

### 3.2. Nuts and Sexual Function

Erectile function and sexual desire are directly influenced by lifestyle factors such as diet through the vascular and nervous systems. For example, Esposito et al. described a direct association between Mediterranean diet adherence and erectile function [72,73], but few authors have studied the most relevant food groups. One early prospective study on nuts involved 17 male patients with erectile dysfunction in an intervention provided with 100 g of pistachios per day for 3 weeks. The authors demonstrated for the first time that a pistachio-supplemented diet improved several of the scores in the International Index of Erectile Function (IIEF), including those related to sexual desire, orgasmic function, and erectile function, among others [74]. However, this was a non-randomized clinical study with outcomes measured before and after the intervention only, which detracts from its quality and casts doubt on the findings. On the other hand, using data from the FERTINUTS trial, Salas-Huetos et al. demonstrated that 60 g day^−1^ of mixed nuts (walnuts, hazelnuts, and almonds) during 14 weeks positively modulated erectile function and sexual desire scores [75]. However, these studies failed to show that these improvements resulted from changes in peripheral levels of nitric oxide or E-selectin, two of the main endothelial function markers. Highlighting the limitations of this research, the authors called for equivalence trials with defined primary outcomes to demonstrate these effects.

### 3.3. Future Research on Nuts and Reproductive Health

Although investigations have begun in this fascinating area, questions remain largely open. In general, more observational studies of good quality and RCTs with larger sample sizes and well-defined inclusion/exclusion criteria are needed to make any recommendations for the general population. We need a better understanding of the mechanisms of action that modulate fertility status and sexual function. Consuming nuts may help to improve the main parameters of semen quality, but there are no current RCTs addressing the effect of paternal and maternal nut consumption and fecundability outcomes. Well-designed intervention and prospective studies in preconception cohorts will help understand the role of nuts in fecundability rates.

## 4. Nuts as Components of Healthy Dietary Patterns

Research involving nuts that addresses the pressing needs of environmental sustainability and novel areas of health, such as reproductive health, both come back to the question of how nuts fit within healthy dietary patterns. This level of research is also evolving, with particular implications for methodological development and translation to policy and practice. While there is a strong history of cultural use of nuts in the human diet, particularly in the Mediterranean regions [1], their broad inclusion in dietary guidance reflects advances in nutrition science. In the last century, there was a focus on nutrients as the basis for providing this guidance [2], but this has evolved to dietary patterns as the burden of disease has shifted to chronic lifestyle-related disease. These non-communicable diseases have multiple and interacting dietary determinants, which best reflect a pattern of food consumption and the synergy that exists between nutrients in foods and foods in a diet [4]. Dietary patterns are now listed among research priorities in a number of authoritative nutrition-related areas, including the US National Institutes of Health “https://dpcpsi.nih.gov/onr/strategic-plan (accessed on 12 February 2023)”, The Australian Academy of Science “https://www.science.org.au/files/userfiles/support/reports-and-plans/2019/2019-nutrition-decadal-plan.pdf (accessed on 12 February 2023)”, and the European collaboration reflected in the EAT–Lancet papers [9].

As naturally occurring plant foods, nuts have a unique nutritional composition characterized by significant proportions of unsaturated fatty acids, fiber and phytosterols, key micronutrients (such as vitamin E and selenium), and polyphenols. Importantly, this reflects the biochemistry of the nut as a living organism, with an interdependence of the nutrients contained therein [76]. Not surprisingly, research has shown that nuts form part of healthy dietary patterns. This last section of the review outlines the research on nuts in healthy dietary patterns, issues relating to their positioning, and directions for future research.

### 4.1. Nuts in Healthy Dietary Patterns

Nut consumption is a key component of numerous dietary patterns known to be associated with a range of health benefits [77]. For instance, nuts feature prominently in diet quality indices such as the Healthy Eating Index (HEI), Alternate Healthy Eating Index (AHEI), and Dietary Approaches to Stop Hypertension (DASH) score, all of which have been associated with a significant reduction in risk of all-cause mortality and incidence of chronic diseases such as (CVD), type 2 diabetes, and cancer [78]. The Mediterranean diet, which has been consistently associated with reduced risk of chronic diseases [79], has regular consumption of nuts as a key feature. Furthermore, examination of a posteriori dietary patterns identified in prospective cohort studies observed that ‘prudent diets’ were associated with reduced risk of coronary heart disease and included a range of beneficial foods, including nuts [80].

Clinical trials have confirmed the beneficial effects of including nuts as components of healthy dietary patterns. For example, the PREDIMED trial, which examined the effect of a Mediterranean diet supplemented with either mixed nuts or olive oil, found a range of health benefits, including reduced incidence of cardiovascular events, when compared to advice on a low-fat diet [62]. A network meta-analysis comparing the effects of consumption of foods and markers of disease in RCTs found that, of the food groups examined, increased consumption of nuts, legumes, and whole grains resulted in the greatest improvements in intermediate risk markers for CVD, nuts being particularly beneficial for reducing LDL cholesterol [81]. Similarly, the inclusion of 30 g of walnuts a day in addition to an interdisciplinary intervention (inclusive of dietary support) for 12 months was found to result in greater weight loss in overweight participants compared to a control diet [82]. These findings are particularly relevant given that nuts are an energy-dense food, with consumers reporting concern regarding the effects of nuts on body weight [83,84]. However, recent meta-analyses have demonstrated that nut consumption does not result in weight gain or increased abdominal adiposity [85], regardless of whether nuts are advised or not to be substituted for other foods [86]. Taken together, these results suggest that nut consumption plays an important role in healthy dietary patterns, with no adverse effects on body weight.

Given the recognized importance of nuts in healthy dietary patterns, regular consumption of nuts is recommended in dietary guidelines globally [87]. While some dietary guidelines [88,89] classify nuts as a food group, many others categorize them with other foods, typically either protein foods [90,91,92,93,94,95] or fats and oils [96,97,98], and some guidelines include nuts in both food groups [99,100,101]. Quantitative recommendations for nut consumption vary between guidelines and appear based on recommendations for the food group, which includes nuts, although the serving size provided in guidelines typically ranges from 15 to 30 g. While approaches to food categorization tend to reflect the protein and fat composition of nuts, variations in food group allocations present challenges when comparing population intakes to recommendations.

Despite the current inclusion in dietary guidelines, population intakes do not appear to meet recommended levels for nut consumption. The 2017 Global Burden of Disease Study noted that global consumption of nuts was approximately 12% of the optimal intake of nuts and seeds (considered to be 21 g per day) [10]. Results from national surveys similarly highlight a common issue of low nut consumption. For instance, a secondary analysis of a subset of the 2005–2018 National Health and Nutrition Examination Survey (NHANES) from the United States of America found 12.9% of adult males and 9.1% of adult females met recommendations to consume 30 g or more of nuts per day [102]. Analysis of the 2011–2013 National Nutrition and Physical Activity Survey in Australia found 5.6% of individuals consuming nuts met the recommendation to eat 30 g of nuts per day [103]. Of note, under 40% of Australians reported consuming nuts during the survey, despite nut consumption including nuts in mixed dishes such as breakfast cereals or muesli bars. Similarly, the European Prospective Investigation into Cancer and Nutrition (EPIC) study found that on the day of the 24 h recall, less than 30% of respondents consumed nuts from any source, although it should be noted that nut intake did vary substantially among countries [104]. These results suggest that population intakes globally do not match current recommendations for nut consumption and highlight the need for increased nut consumption as part of a healthy dietary pattern.

### 4.2. Positioning Nuts in the Diet

Translating the evidence supporting nuts in healthy dietary patterns requires an understanding of nut consumption with other foods and within meals. The lack of congruence between recommendations and actual intakes suggests more work is required in that area. Nuts can serve as snack foods, and while research has exposed their impact on diet quality when substituted for poor-quality snacks [105], positioning them as snacks can align them with incidental, non-staple foods. In addition, theoretical positions of nuts in trials of healthy dietary patterns does not always translate to their inclusion as a staple food. For example, nuts were critical foods in the PREDIMED study, which demonstrated the CVD-preventive effects of the Mediterranean diets supplemented with extra-virgin olive oil or mixed nuts [62], but were less obvious in a study of staple foods in the context of food insecurity in the USA [106]. On the other hand, nuts are emerging as key foods in sustainable diets [107].

Behind this issue lies the question of collecting and managing research data on nuts. One suggestion is to create a separate food group of nuts, possibly with seeds as contemporaries [108]. This would clarify measurement in dietary surveys, albeit with a need to address the name of the group, serving size, and frequency of consumption. There are also implications for how nuts might be included in dietary indices that evaluate diet quality and how they might fit within a cuisine pattern. Even if this were the case, there have been major shifts away from naming food groups in terms of actual foods, with a greater focus on degree of processing.

The NOVA system of food categorization [109] places nuts favorably in the desired ‘unprocessed or minimally processed’ food category. This position is consistent with accompanying research that indicates a risk to cardiovascular health with ultra-processed foods, explainable through the loss of natural food synergy, displacement of healthy foods, and high content of saturated fat, sugar, and salt [110]. There is substantial debate on this methodological development, arguing the issue of misclassification with the NOVA system, the lack of associated conventional research, and the adequacy of current nutrient scoring systems [111]. Nevertheless, the minimally processed food category is consistent with staple foods recommended in dietary guidelines [112], which may assist in better compliance. The debate has brought into question not just food groupings, but food classification systems in general used in the review of dietary guidelines [113]. Whether these are nutrient-based rating or scoring systems, or food categories based on processing (such as NOVA), or dominant nutrient contribution (as in dietary guidelines), there is wide variation in agreement in the way they present the health potential of individual foods.

Healthy dietary patterns tend to be investigated using forms of diet quality indices or scores (for example, Mediterranean diet scores) [114]. They address the whole of diet relationships with health outcomes, such as CVD risk, and take various approaches to the consumption of foods and nutrients and/or dietary patterns/cuisines. It is important to note, however, that they also serve various purposes: from health promotion activity to food labeling requirements and from measuring relationships in observational studies to effects seen in intervention trials. The positioning of nuts in instruments that address dietary patterns would need to take into consideration the purpose of the research activity.

### 4.3. Future Research on Nuts in Healthy Dietary Patterns

The ability to discern the impact of nuts in a dietary pattern, as with any food, will depend on how they are categorized and treated in the analysis [77]. Of the two main analytical approaches: a posteriori (looking for groups, identifying and naming patterns), or a priori (using pre-determined dietary patterns), those using an Index (a priori approach) are the commonest, followed by Factor or Principal Component Analysis (a posteriori approaches) [115]. This is also a developing area, with varying applications and reporting systems, as well as attention to foods and/or nutrients. Building an argument that deals with the interrelationship between foods and nutrients, however, strengthens the evidence of food effects. For example, using Principal Component Analysis in a study of baseline relationships between nutrients, foods, and dietary patterns with blood pressure, the dietary pattern categorized by nuts, seeds, fruit, and fish was significantly associated with lower blood pressure and with lower sodium:potassium ratio (a nutrient ratio related to blood pressure) [116]. In this case, the analyses based on healthy foods and intervening nutrients produced congruent outcomes.

Building the evidence base for nuts in healthy dietary patterns continues to rely on epidemiology to provide evidence of associations, clinical trials to demonstrate effects, and experimental laboratory studies to expose explanatory mechanisms [4]. Systematic reviews, meta-analyses, and quality assessments add rigor to the process, but this is a dynamic system requiring regular updates, oversight, and dedicated funding [7]. The purpose of building the evidence base will also inform the nature of the research, and this includes a consideration of transitional issues such as consumer communications. For example, research has shown the presence of health claims influences consumer purchasing, but there is more to do around how consumers understand and act on health claims in relation to meal contexts and overall dietary patterns [117].

## 5. Conclusions

Nuts are healthy foods: they are a source of important micronutrients, unsaturated fatty acids, protein, fiber, and plant sterols, and they form part of recognized healthy dietary patterns. Today, however, there is an imperative to review the impact of dietary patterns on the environment. This has led to a shift to plant-based diets, where nuts emerge as a significant source of protein. Health perspectives see nuts as a minimally processed and sustainable food, but research at the production level is evolving. Given their high nutritional value, environmental research is likely to drive better nut production methods in varying climate conditions. Nuts remain an important contributor to human health, with the mechanisms of action explained in terms of the nutrients they deliver. Studies have linked nut consumption to better blood lipoprotein profiles and lower CVD risk, but early research is now indicating possible beneficial effects of nut consumption at the other end of the life spectrum, namely reproductive health. This is a novel and interesting area of new research with many questions open for further investigation. Whether we consider the production of nuts or their consumption, the position of nuts in the dietary pattern remains an issue. The ultimate effects of food on health are the results of multiple interactive factors, so where nuts fit within dietary patterns is a significant consideration for research translation. There are implications for research methodologies, including categorization within food groups and inclusion in Healthy Dietary Indices.

One of the most significant issues for research translation is that the consumption of nuts in many jurisdictions across the globe does not meet evidence-based recommendations. New areas of research, such as reproductive health discussed here, may help to increase the recognition of nuts as important foods in the diet. Likewise, their role in plant-based diets aimed at addressing environmental as well as health concerns may be casual. While continuing to build the evidence base on the health benefits of nuts, a focus should remain on methodology affecting the positioning of nuts in dietary assessment instruments, which may, in turn, influence forms of communication to consumers.

In dietary surveys, a separate category of nuts (and possibly seeds) may address the problem, as could the inclusion of nuts in healthy diet indices. Translational targets also require clarity of purpose in research. The nutrition science community recognizes the diversity of research methods to ‘advance discovery, interpretation and application of knowledge’ [118]. This includes an appreciation of how different layers of knowledge create the evidence base that enables appropriate (dietary) recommendations. In research on the health benefits of nuts, expanding the scope of interest to health throughout the lifecycle, especially in the area of reproductive health, and integrating research on environmental issues and sustainable diets represent very positive ways forward.

## Data Availability

Not applicable.
